# A biomechanical comparison of steel screws versus PLLA and magnesium screws for the Latarjet procedure

**DOI:** 10.1007/s00402-021-03898-w

**Published:** 2021-04-30

**Authors:** Benjamin Bockmann, E. Jaeger, L. Dankl, W. Nebelung, S. Frey, W. Schmölz, T. L. Schulte

**Affiliations:** 1 Department of Orthopaedics and Trauma Surgery, St. Josef Hospital, Ruhr University Bochum, Gudrunstrasse 56, 44791 Bochum, Germany; 2 Department of Orthopaedics and Traumatology, Medical University of Innsbruck, Innsbruck, Austria; 3 OPND-Praxisklinik, Plange Mühle 4, Düsseldorf, 40221 Germany; 4 Department of Orthopedic, Trauma- and Handsurgery, Florence-Nightingale-Hospital, Düsseldorf, Germany; 5 Department of Sports Orthopaedics, St. Vinzenz Hospital, Schloßstraße 85, Düsseldorf, Germany

**Keywords:** Glenoid deficit, Coracoid process, Latarjet procedure, Fixation, Shoulder instability, Biodegradable

## Abstract

**Introduction:**

The fixation of the coracoid process onto the glenoid is an important step of the Latarjet procedure, and implant-associated complications are a relevant and severe problem. This study compares the fixation strength and failure mode of two biodegradable materials with stainless-steel screws.

**Methods:**

24 Fresh-frozen cadaveric scapulae were divided into three groups of equal size and received a coracoid transfer. Cadavers were matched according to their bone mineral density (BMD). In group 1, small-fragment screws made of stainless steel were used. In the second group, magnesium screws were used, and in the third group, screws consisted of polylactic acid (PLLA). A continuously increasing sinusoidal cyclic compression force was applied until failure occurred, which was defined as graft displacement relative to its initial position of more than 5 mm.

**Results:**

At 5-mm displacement, the axial force values showed a mean of 374 ± 92 N (range 219–479 N) in group 1 (steel). The force values in group 2 (magnesium) had a mean of 299 ± 57 N (range 190–357 N). In group 3 (PLLA), failure occurred at 231 ± 83 N (range 109–355 N). The difference between group 1 (steel) and group 2 (magnesium) was not statistically significant (*P* = 0.212), while the difference between group 1 (steel) and group 3 (PLLA) was significant (*P* = 0.005).

**Conclusion:**

Stainless-Steel screws showed the highest stability. However, all three screw types showed axial force values of more than 200 N. Stainless steel screws and PLLA screws showed screw cut-out as the most common failure mode, while magnesium screws showed screw breakage in the majority of cases.

**Evidence:**

Controlled laboratory study.

## Introduction

Glenoid bone loss is a common cause of failed Bankart repair in the shoulder [1]. If bone loss exceeds 15% of the glenoid surface in patients with recurrent anteroinferior shoulder instability, the condition can be considered critical and osseous reconstruction is recommended [2]. One of the most frequently used techniques for restoring the native shape of the glenoid is to transfer the coracoid process to the anteroinferior glenoid [3, 4]. According to current research, the Latarjet procedure accounts for 3.4% of all stabilization procedures in the United States between 2007 and 2015, with a significant annual increase of 15.4% annually [5]. However, reliable fixation of the coracoid process is essential to achieve good clinical results for all techniques [6, 7], and dislocation of the bone graft accompanied by screw breakage has been identified as a severe complication [8–10]. A systematic review by Griesser et al. [11] found implant-associated revisions in 2% of all patients.

Another recent development is the use of biodegradable materials that dissolve during the initial months after surgery [12, 13]. One of the expected advantages of these materials is that they avoid soft tissue irritation of the surrounding tissue on the one hand and cartilage damage of the humeral head on the other.

The aim of this study was to evaluate the strength of different fixation methods for the coracoid process applicable to those situations in which the coracoid graft is directly loaded, through either graft malpositioning, intense early rehabilitation, or shoulder trauma. The null hypothesis was that no difference could be found between conventional steel screws and the two biodegradable materials.

## Materials and methods

### Cadavers and materials

Upon commencing the study, a pre-test power analysis was performed based on the results for a maximum cyclic displacement of partially threaded solid 4.0-mm cancellous screws with bicortical fixation and fully threaded solid 3.5-mm cortical screws with bicortical fixation in the study performed by Shin et al. [14]. Based on a power of 50% and a probability of a type-I-error of 0.2, 7 cadavers in each group were determined as a group size [15].

Twenty-four fresh-frozen cadaveric scapulae were acquired from commercial vendors and assigned to three groups with 8 cadavers per group. The bones consisted of 16 male and eight female specimens (group 1: 5 male, 3 female; group 2: 4 male, 4 female; group 3: 7 male, 1 female) that were donated pairwise by eight male and four female donors. Their mean age was 54 years (range 28–64 years, group 1: 55 ± 10 years, group 2: 53 ± 14 years, group 3: 54 ± 12 years), and all specimens with previous shoulder surgery or previous injuries to the bone were excluded.

Prior to testing, quantitative computed tomography (qCT) scanning (LightSpeed VCT, GE Healthcare, Chicago, USA; image size 512 × 512, slice thickness 625,000 μm) was performed on all specimens to detect osseous lesions, cysts, or fractures. In addition, the bone mineral density (BMD) was evaluated using dedicated software (ImpaxEE, Agfa HealthCare GmbH, Bonn, Germany). On the basis of the study by Shin et al. [14], three subchondral BMD values were measured 5, 7, and 9 mm medially of the glenoid surface using parasagittal views by a single, experienced observer. For each specimen, the mean of the three values was calculated and taken as the BMD value.

On the basis of their BMD values and age, the specimens were assigned to three groups:

- In Group 1, a Latarjet procedure was performed and the coracoid process was attached to the deficient glenoid using two small-fragment screws (Reference number 204.836, shaft ⌀ 2.7 mm, shaft thread ⌀ 3.5 mm, DePuy Synthes GmbH, Oberdorf, Switzerland). This group represented the clinical standard and was therefore considered the control group.
- In Group 2, two magnesium screws were used (Magnezix® CS 3.2, article number 1032.036, Syntellix AG, Hannover, Germany; shaft ⌀ 2.4 mm, cannulation ⌀ 1.3 mm, head thread ⌀ 4 mm, shaft thread ⌀ 3.2 mm, with different pitches allowing interfragmentary compression).
- In Group 3, the coracoid was attached with two solid enhanced polylactic acid (PLLA) screws (Bio-Compression Screws, article number AR-5025B-26, Arthrex, Naples, Florida, USA; shaft ⌀ 3 mm, shaft thread ⌀ 3.7 mm, 2.7-mm screwdriver insertion).

### Surgical technique

The scapulae were stored at − 20 °C and thawed at room temperature for 12 h before dissection and testing.

**Fig. 1:**
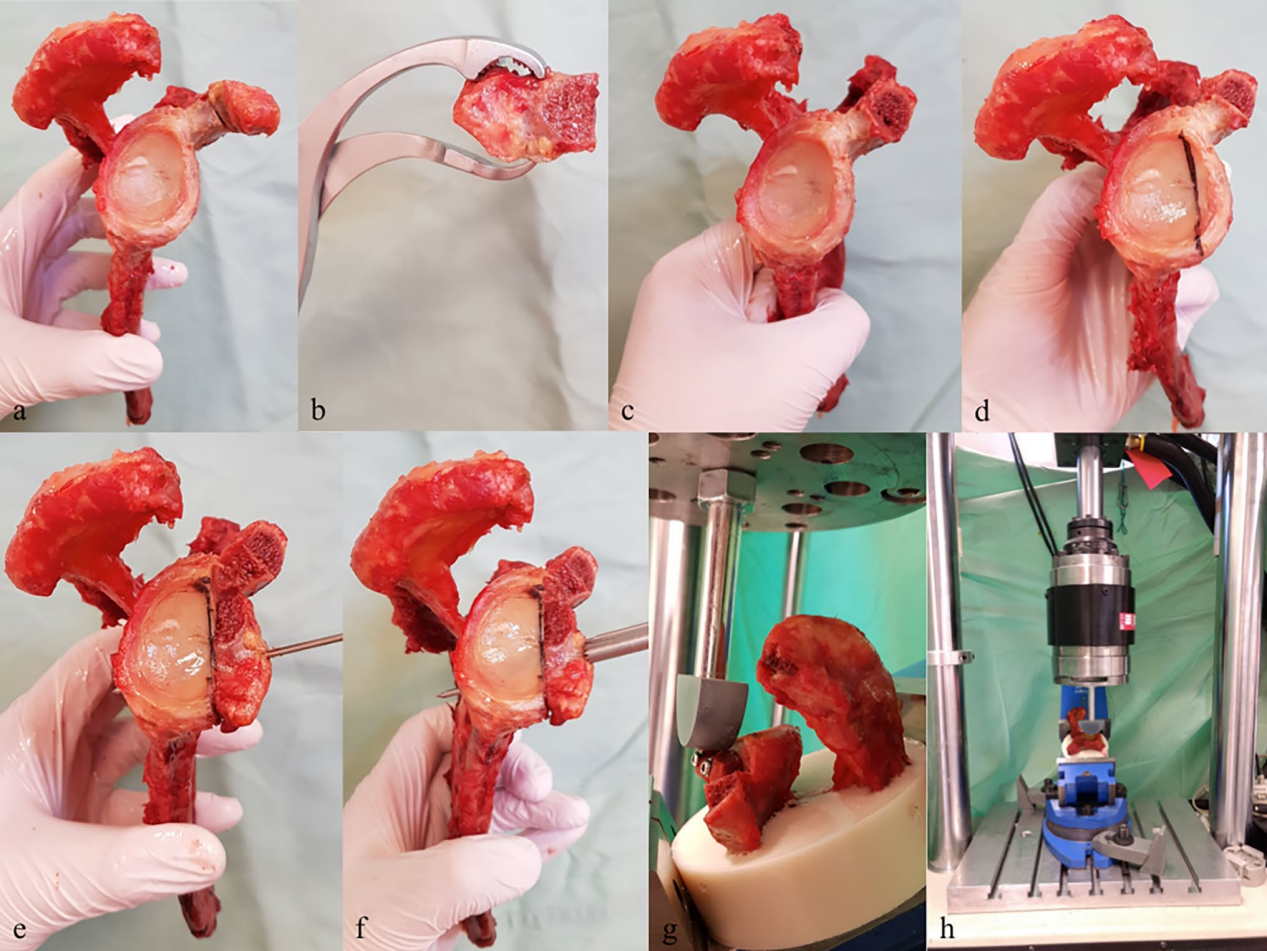
Example preparation of a cadaver. **a** All soft tissue was removed. **b**, **c** The coracoid was osteotomized at 25 mm from the tip, using an oscillating surgical saw. **d** The width of the glenoid defect was evaluated using a metal ruler and drawn onto the bone with a waterproof pen. **e** A 1-mm K-wire was placed at the center of the graft, and the graft was temporarily attached to the glenoid defect. **f** A parallel drill guide (article number AR-5024, Arthrex, Naples, Florida, USA) was used to ensure identical screw placement for all specimens, taking the first K-wire as the reference point. **g** The embedded scapulae were fixed at a 30° angle to the actuator of the servohydraulic testing machine. **h** The overall test set-up, including the servohydraulic testing machine

The adjacent soft tissue, including rotator cuff muscles, ligaments and fat tissue, was removed using surgical scalpels (Fig. 1a). The scapulae were then mounted in an anatomic stand, and the coracoid was osteotomized at 25 mm from the tip using an oscillating surgical saw, as described by Shin et al. [14] (Fig. 1b, c). In the next step, the aspect of the graft that was later attached to the glenoid was carefully decorticated using an oscillating saw. A graft thickness of at least 7 mm was maintained in all coracoid fragments [14]. Bone loss of 25% was simulated for each scapula individually on the basis of the qCT scan results, using the formula and technique described by Bhatia et al. [16]. The width of the glenoid defect was evaluated with a metal ruler and drawn onto the bone with a waterproof pen. The defect was then created using the oscillating saw (Fig. 1d). Hence, the final width of the construct was determined by two components: first, the deficient glenoid, which had lost 25% of its width, and second the width of the graft, which was at least 7 mm in all constructs.

A 1-mm K-wire was placed in the center of the graft and was temporarily attached to the glenoid defect (Fig. 1e). The coracoid was positioned using the congruent arc configuration proposed by de Beer et al. [17], so that the dished undersurface of the coracoid process later was in line with the glenoid surface. Care was taken to ensure that the coracoid fragment was placed flush with the glenoid surface and that the inferior concavity of the coracoid process later formed the articular aspect of the glenoid (Fig. 1e). Afterward, a parallel drill guide (article number AR-5024, Arthrex, Naples, Florida, USA) was used to ensure identical screw placement for all specimens [18], using K-wire as the reference point (Fig. 1f).

**Fig. 2:**
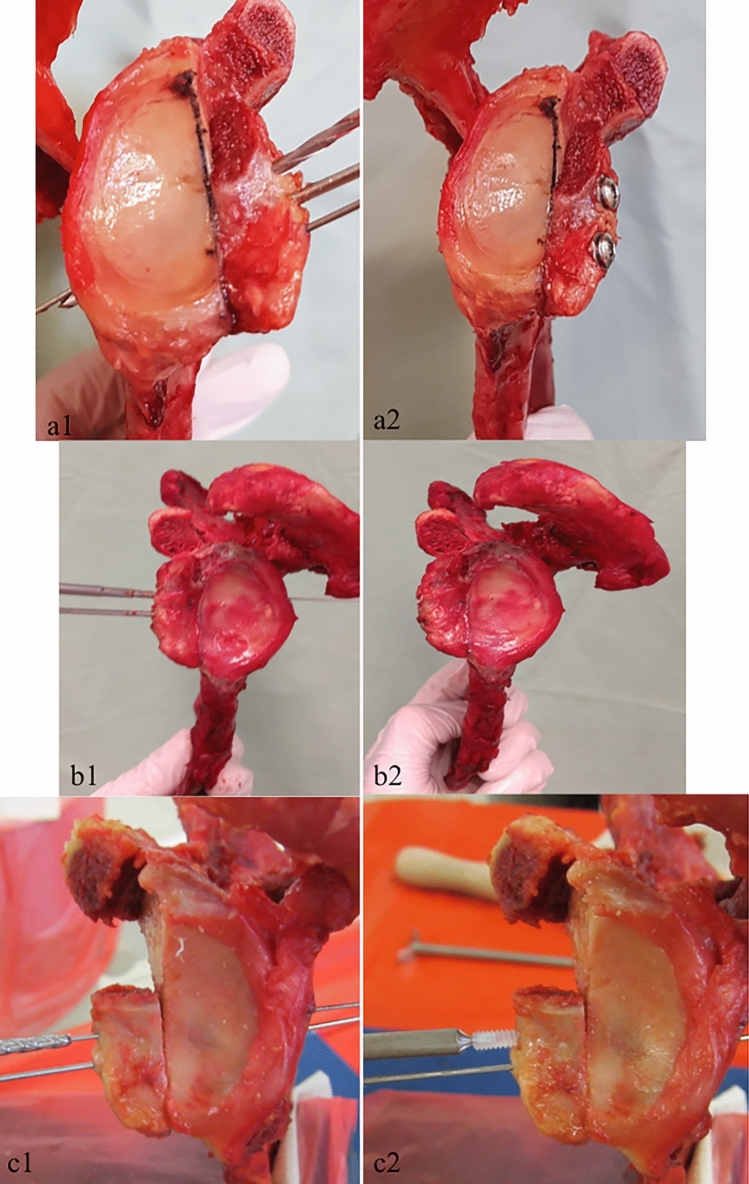
Installation of the three different screw types. In group 1 (steel), the graft was secured with a central K-wire (**a1**). A 3.5-mm glide hole and a 2.7-mm thread hole were created using cannulated drills, in the AO technique. The graft was placed flush to the glenoid surface (**a2**). In group 2 (magnesium), a cannulated drill was used to create a 2.5-mm tunnel (**b1**). A 3.5-mm countersink was then used to create a hole for the screw head. The screw was then placed using the K-wire as guidance. Again, two screws were placed to fixate the graft flush to the glenoid surface (**b2**). In group 3 (PLLA), a K-wire was again used as guidance for the superior screw (**c1**). The superior hole was drilled using the dedicated drill until a laser mark was reached. A dilator tap was then used to create a screw thread inside the drill hole. Finally, the screws were inserted using the Bio-Compression screwdriver (**c2**)

In group 1, 3.5-mm small-fragment screws made of stainless steel were used. The length of all screws was 36 mm, in accordance with the findings of McHale et al. [19], and the screws were applied bicortically as proposed by Schmiddem et al.[20]. Using the drill guide, two parallel 1.0-mm K-wires were placed gradually superior and inferior to the first K-wire (Fig. 2a1). The two screws were placed under interfragmentary compression using the standard AO lag screw technique (Fig. 2a2). For the superior screw, the anterior cortex of the coracoid graft was overdrilled with a 3.5-mm standard cannulated drill, and the posterior glenoid cortex with a 2.7-mm drill. Afterward, the respective K-wire was removed and the first small-fragment screw was placed with a flat washer. Mild compression was applied with two fingers. The same technique was then used to place the inferior screw. Finally, the central K-wire was removed.

In group 2, two Herbert-type screws made of magnesium were used. The screw material consists of a magnesium alloy (based on MgYREZr) and contains > 90% magnesium. Again, all of the screws were 36 mm long and were installed bicortically. For the application, two K-wires were placed and drill holes were installed with the dedicated 2.5-mm drill bit and 3.5-mm countersink, as described above (Fig. 2b1). Finally, the two screws were placed with the recommended, cannulated screwdriver using the K-wires in the screw holes as guidance. Again, only mild compression was applied (Fig. 2b2).

In group 3, two PLLA screws were used. These screws are made of enhanced polylactic acid and have a conical shape. The maximum available screw length (26 mm) was used. The application differed from that used in group 1 (steel) and 2 (magnesium), as these screws are not applied using the lag screw technique. Using the drill guide, two holes were drilled using the dedicated drill until a laser mark was reached, indicating that the drill hole was at the right depth (Fig. 2c1). Then, a dilator tap was used to create a screw thread within the drill hole. Finally, the screws were inserted using the Bio-Compression screwdriver (Fig. 2c2).

### Embedding and biomechanical testing

Once the Latarjet procedure had been performed, each scapula was shortened at the level of the suprascapular notch. The cutting line was parallel to the glenoid surface.

The constructs were then embedded in epoxide resin (RenCast, Huntsman Advanced Materials, Basel, Switzerland) up to 1 cm below the graft. Biomechanical testing was carried out using a biaxial servohydraulic material testing machine (MTS 858 Mini Bionix II; MTS, Eden Prairie, Minnesota, USA) (Fig. 1g, h). Correct bicortical screw placement was confirmed using conventional radiographs.

Biomechanical testing was performed in accordance with a previously published model [14, 21, 22]. Thus, a special loading head with a radius that simulates the average curvature of a humeral head (25 mm) was created to load only the coracoid graft and simulate compression of the construct by the humeral head in abduction and external rotation. Embedded scapulae were fixed at a 30° angle to the actuator of the servohydraulic testing machine (Fig. 1g). The loading head was aligned with the coracoid graft and preloaded at 1 N. The graft was then loaded with a continuously increasing sinusoidal cyclic compression force (1 Hz) with a lower limit of 50 N and a constantly increasing upper load limit starting from 100 N. The upper limit increased by 0.1 N after each cycle until structural failure was reached. Structural failure was defined as fixation failure or 5-mm displacement of the loading piston relative to its initial starting position [14].

### Statistical analysis

The test data were recorded using the testing machine’s dedicated software and later organized in a Microsoft Excel spreadsheet. Statistical data analysis was carried out using IBM SPSS Statistics for Windows, version 24.0.0.1 (Armonk, New York, USA: IBM Corporation). Kolmogorov–Smirnov tests were used to test for normal distribution. If normal distribution was present, one-way analysis of variance (ANOVA) was performed to test for the significance of differences in basic parameters (e.g., BMD) between the groups. To evaluate the significance of axial displacement in detail, a post hoc test (Bonferroni) was carried out. For all tests, *P* values lower than 0.05 were defined as significant.

## Results

Statistical evaluation using the Kolmogorov–Smirnov test showed that BMD values in the overall cohort were normally distributed (*P* = 0.200), with a mean of 163.2 g/cm^3^ ± 44.32 (range 91.3–272.1 g/cm^3^). The BMD values for the respective groups were 165.6 g/cm^3^ ± 54.8 in group 1 (steel), 162.2 g/cm^3^ ± 39.8 in group 2 (magnesium), and 161.9 g/cm^3^ ± 43.0 in group 3 (PLLA). The differences between groups 1 (steel) and 2 (magnesium), and between groups 1 (steel) and 3 (PLLA) were not significant (*P* = 0.887 and *P* = 0.881, respectively).

No preexisting bone lesions were noted in the qCT scans. After screw placement, all of the radiographs confirmed intact constructs, with no screw breakages or graft fractures.

**Fig. 3:**
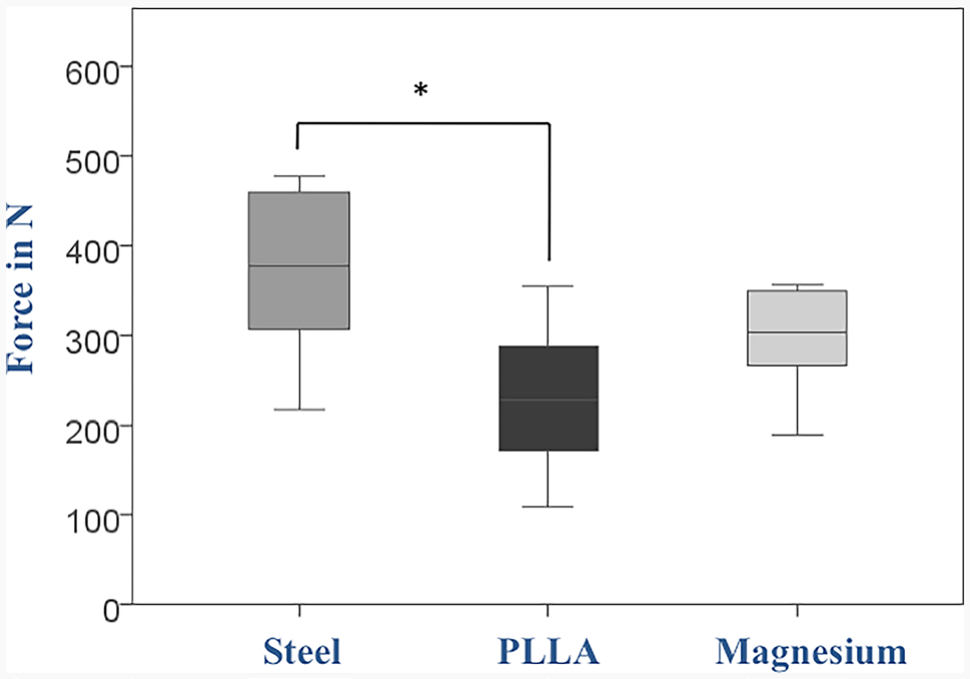
Force values at 5 mm displacement of the graft relative to its initial position

At 5 mm displacement, the axial force values showed a mean of 374 N ± 92 in group 1 (steel) (range 219–479 N; Fig. 3). The force values in group 2 (magnesium) had a mean of 299 N ± 57 (range 190–357 N). In group 3 (PLLA), failure occurred at 231 N ± 83 (range 109–355 N). The difference between groups 1 (steel) and 2 (magnesium) did not reach statistical significance (*P* = 0.212), but the difference between group 1 (steel) and group 3 (PLLA) was significant (*P* = 0.005).

**Fig. 4:**
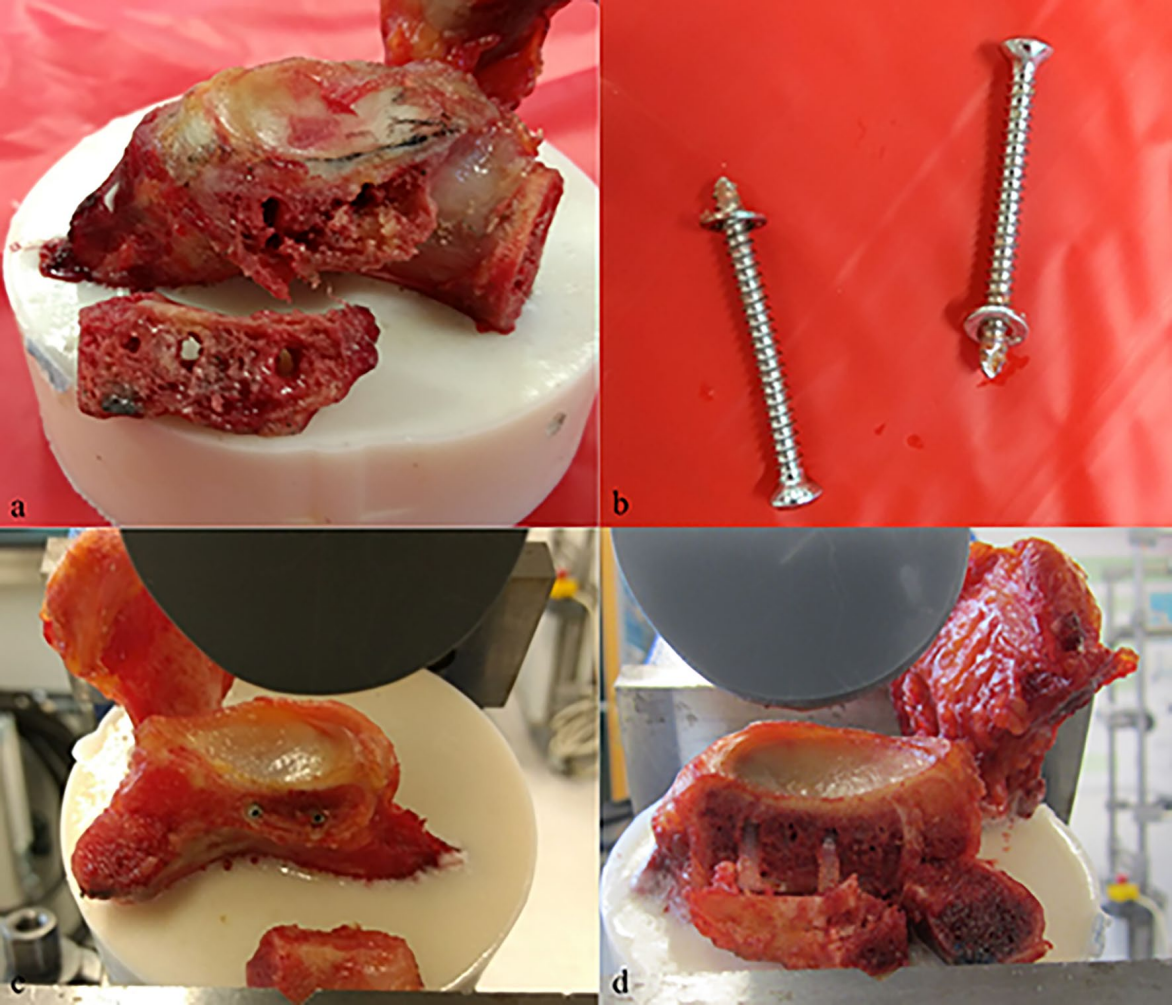
Different failure mechanisms. **a** Screw cut-out in group 1 (steel). **b** The screws show signs of deformation, but remain intact. **c** Screw breakage in group 2 (magnesium). The screw tips are still in the glenoid. **d** Screw cut-out in group 3 (PLLA). Again, the screws show signs of deformation, but remain intact

Three failure modes were observed: screw cut-out, with both the graft and the implants remaining intact (Fig. 4a, b, d); graft breakage; and screw breakage (Fig. 4c). In group 1 (steel), all eight scapulae showed screw cut-out with intact screws. In group 2 (magnesium), five specimens showed screw breakage and two had screw cut-outs; one specimen showed failure with one loosened screw and one broken screw. In group 3 (PLLA), six screws had cut-outs and two specimens showed graft fractures.

Three constructs failed before 5-mm displacement of the graft was observed: one specimen in group 3 (PLLA) showed graft breakage at a displacement of 4.5 mm with an axial force of 208 N. Failure occurred in two specimens in group 2 (magnesium) due to screw breakage in the implanted materials: one at 3.2-mm displacement with 354 N, the other at 4.6-mm displacement with 357 N.

## Discussion

The central finding of this study is that stainless-steel screws showed the greatest primary fixation strength when a direct load is applied to the graft. However, both of the biodegradable materials tested showed a failure of the construct at more than 200 N. Between stainless-steel screws and magnesium screws, no significant difference was found.

In general, bioabsorbable materials have been shown to be potentially associated with local tissue damage. Plaass et al. [12] conducted a study in which 45 correction osteotomies were performed in hallux valgus cases using magnesium screws similar to those used in the present study. In their study, corrosion effects resulting in hypodense bony cavities around the implant were observed. Radiolucencies around the screw bodies were also noted and interpreted as indicating a retreat of calcified bone. The development of these radiolucencies is not fully understood. One possible explanation is the formation of hydrogen, which develops during the corrosion of magnesium. Although the overall clinical results in the cohort were good, this aspect has yet to be investigated.

In relation to PLLA-based implants, similar to the material used in group 3 in the present study, Böstman et al. [23] presented a comprehensive cohort study including 2528 patients. The authors analyzed a subgroup of patients who received PLLA screws for fracture treatment and found that one patient out of 491 (0.2%) had local inflammatory signs that were thought to be caused by the implant. This adverse event was observed 4.3 years after treatment for an ankle fracture. Although this is a rather low percentage, the authors concluded that the biocompatibility of the substance requires better clinical analysis.

One disadvantage of steel screws is that in case of insufficient union or failure of the coracoid graft, remaining screws are large irritating objects that can damage the surrounding anatomical structures and lead to revisions [21]. In a systematic review that analyzes the most frequent complications after the Latarjet procedure, Griesser et al. [11] found 45 cases of unplanned hardware removal among a total of 1881 patients. Upon that, the clinical implication of screw dislocation is immense. When supernatant screw heads irritate the surrounding subscapularis muscle, shoulder pain in adduction and external rotation can impair clinical overall results and patient satisfaction [24]. These concerns have further enhanced the design of biodegradable materials with a lower risk of soft-tissue damage and the need for revision.

A review of the current biomechanical literature reveals a lack of data concerning the use of biodegradable materials for the Latarjet procedure. In the studies that are available, comparable pull-out strengths can be found with regards to our results from groups 2 (magnesium) and 3 (PLLA). Weppe et al. [25] compared the primary stability of biodegradable interference screw fixation of a coracoid bone plug and a conventional Latarjet–Patte coracoid transfer with bicortical screw fixation. They reported pull-out strengths of 110 N for the interference screw and 202 N for the control group, whereas the interference screw constructs ranged from 95 to 170 N. 10 cadavers where used in each group. In the present study, the primary stability of all three test groups (steel, magnesium or PLLA screws) was higher than in the study of Weppe et al. However, direct comparison of these two studies is hardly possible, because of the substantial higher age of specimens used by Weppe et al., (mean age 87 years, vs present study 54 years).

In another study, Shin et al. [14] compared different non- resorbable screw types for fixation of the coracoid. They defined graft displacement of more than 5 mm as a failure and reported failure loads between 495 and 562 N for different screws types. Their failure loads were higher than in the present study. However, they investigated only non-resorbable screws and specimens that might have had an better bone quality.

The authors are aware of the limitations of the present study. Firstly, as a controlled laboratory study, the results merely analyzed the primary stability of the constructs. Other important aspects such as the bone–implant interaction cannot be implemented in cadaver tests and require further examination. Secondly, all soft tissue was removed from the bones during testing. Particularly in the shoulders, passive and active stabilizers such as the rotator cuff and the capsule have an important influence on glenohumeral stability. However, the experimental setting was unable to take these mechanisms into account. Finally, it should be mentioned that the manufacturers of both materials used in group 2 (magnesium) and 3 (PLLA) do not yet officially recommend these implants for use in Latarjet procedures. The application of these implants for the tested purpose must therefore be regarded as off-label use.

## Conclusion

Stainless steel screws showed the highest stability. However, all three screw types showed axial force values of more than 200 N. Stainless steel screws and PLLA screws showed screw cut-out as the most common failure mode, while magnesium screws showed screw breakage in the majority of cases.
